# Supplementary material for: Anxiety, PTSD, and stressors in medical students during the initial peak of the COVID-19 pandemic
Source: PLoS One. 2021 Jul 29;16(7):e0255013. doi: 10.1371/journal.pone.0255013 (PMC8320894; doi:10.1371/journal.pone.0255013)
Supplement: S1 Table — (DOCX) [file pone.0255013.s002.docx]

**S2 Table. Eligible clinical student populations by medical school**

| School | Classes Eligible | N |
| --- | --- | --- |
| University of California, San Francisco* | 2020  2021  2022 | 162  186  208 |
| University of California, Irvine⧫ | 2020  2021 | 104  104 |
| University of Illinois⧫ | 2020  2021 | 316  334 |
| Ohio State University⧫ | 2021  2022 | 201  209 |
| Tulane University⧫ | 2020  2021 | 195  191 |
| Hofstra University* | 2020  2021  2022 | 99  101  101 |

*Schools which begin clinical rotations in the second year of medical school

⧫Schools beginning clinical rotations in the third year of medical school

Curricula vary by medical school in the United States. Students in clinical years were deemed eligible for the survey if they had begun clinical rotations by the time of survey administration. Class years were based on the class that a student started medical school in, independent of any planned or unplanned time off; for an MD-PhD student starting in the class of 2021, for example, they were eligible if they had begun clinical rotations before April 2020 regardless of when they started their research time.
