# Supplementary material for: Anxiety, PTSD, and stressors in medical students during the initial peak of the COVID-19 pandemic
Source: PLoS One. 2021 Jul 29;16(7):e0255013. doi: 10.1371/journal.pone.0255013 (PMC8320894; doi:10.1371/journal.pone.0255013)
Supplement: S2 Table — (DOCX) [file pone.0255013.s003.docx]

**S3 Table. Descriptive statistics for anxiety and PTSD scales**

| GAD-7  How often have you been bothered by the following over the past 2 weeks: | Not at all  N (%) | Several days  N (%) | More than half the days  N (%) | Nearly every day  N (%) |
| --- | --- | --- | --- | --- |
| Feeling nervous, anxious or on edge? | 191 (25.9%) | 310 (42.1%) | 155 (21.0%) | 81 (11.0%) |
| Not being able to stop or control worrying? | 323 (43.8%) | 250 (33.9%) | 103 (14.0%) | 62 (8.4%) |
| Worrying too much about different things? | 223 (30.3%) | 285 (38.7%) | 136 (18.5%) | 93 (12.6%) |
| Trouble relaxing? | 234 (31.9%) | 286 (39.0%) | 134 (18.3%) | 80 (10.9%) |
| Being so restless that it's hard to sit still? | 436 (59.4%) | 174 (23.7%) | 82 (11.2%) | 42 (5.7%) |
| Becoming easily annoyed or irritable? | 244 (33.0%) | 305 (41.3%) | 125 (16.9%) | 65 (8.8%) |
| Feeling afraid as if something awful might happen? | 402 (54.4%) | 210 (28.4%) | 84 (11.4%) | 43 (5.8%) |
|  | | | | |
| Score Range | None (0-4) | Mild (5-9) | Moderate (10-14) | Severe (15-21) |
|  | 292 (39.6%) | 253 (34.3%) | 119 (16.1%) | 70 (9.5%) |

| PC-PTSD-5  Since the start of the COVID-19 pandemic, have you: | Yes  N (%) |
| --- | --- |
| Had nightmares related to the pandemic or thought about the pandemic when you did not want to? | 230 (31.1%) |
| Tried hard not to think about the pandemic or gone out of your way to avoid situations that reminded you of it? | 298 (40.4%) |
| Been constantly on guard, watchful, or easily startled? | 168 (22.8%) |
| Felt numb or detached from people, activities or your surroundings? | 253 (34.4%) |
| Felt guilty or unable to stop blaming yourself or others for the effects of the pandemic or any problems the pandemic may have caused? | 152 (20.6%) |
|  | |
| At least 3 “yes” | 188 (25.4%) |
| At least 4 “yes” | 86 (11.6%) |
