# Supplementary material for: Anxiety, PTSD, and stressors in medical students during the initial peak of the COVID-19 pandemic
Source: PLoS One. 2021 Jul 29;16(7):e0255013. doi: 10.1371/journal.pone.0255013 (PMC8320894; doi:10.1371/journal.pone.0255013)
Supplement: Attachment — Submitted filename: R2R.docx [file pone.0255013.s004.docx]

*PONE-D-21-08270*

*Anxiety, PTSD, and stressors in medical students during the initial peak of the COVID-19 pandemic A mixed- methods study*

*PLOS ONE*

*Dear Dr. Lee,*

*Thank you for submitting your manuscript to PLOS ONE. After careful consideration, we have decided that your manuscript in the current form does not meet our criteria for publication and must therefore be rejected.*

*Specifically:*

*Though the authors have conducted a very interesting study on a very important mental health issue, still there are certain areas with lack methodological clarity. The study design does not appear to be clear as a whole, especially the design of mixed-methods study in the current manuscript.*

*I am sorry that we cannot be more positive on this occasion, but hope that you appreciate the reasons for this decision. However, the authors may consider re-submitting their manuscript after addressing the comments appended below.*

*Yours sincerely,*

*Dr. Arista Lahiri, MBBS, MD*

*Academic Editor*

*PLOS ONE*

*Additional Editor Comments (if provided):*

*The authors have conducted a very interesting study on an important topic pertaining to the mental health of medical students, during the first peak of the COVID-19 pandemic. Though the authors have sequentially elaborated their findings, but overall the study suffers certain limitations:*

*1. It is difficult to comprehend why this study has been identified as a mixed-methods study by the authors. Need justification and further details related to mixed-methods design.*

Thank you—after reexamination, we have removed the term “mixed methods” when reporting our quantitative results and thematic analysis. These changes have been made to the title and methods sections and are highlighted in the tracked changes version of the manuscript.

Our study combines thematic analysis of students’ stated reactions to the significant educational disruption of the pandemic with validated measurements of mental health outcomes, a synthesis that has not been attempted elsewhere. We also used census-based sampling of all eligible students from six medical schools deliberately identified to form a representative profile of regional diversity and COVID-19 prevalence at the time. Finally, our data reflect the burdens on students during the initial peak of the pandemic, when they were faced with both the greatest academic uncertainty and concern for the safety of self and community. These three features make our study a unique contribution to understanding the pandemic’s effects on a subset of future doctors and help us prepare for future pandemics.

*2. The authors need to have a look at their Survey design, particularly the sampling part of their study*

This is a helpful suggestion; to further clarify our sampling, we have created Supplemental Table 2 that shows eligible populations of students at each school. We intentionally sampled medical schools to represent both levels of regional COVID-19 prevalence and the four major geographical regions of the United States. During this pandemic, studies of acute mental health effects have used representative regional sampling to estimate prevalence [28].

In a census sampling approach, we distributed the survey to all eligible students in clinical training years at these schools (Supplemental Table 2 included below). In contrast to convenience sampling via social media used in other studies of medical students during the pandemic [21], we believe census sampling allows a representative cross-section of medical students’ diverse experiences to be constructed.

*3. The qualitative methods if any used is unclear. The qualitative analytical technique also is unclear.*

Thank you, we have removed the term “qualitative” to avoid any confusion. In the Methods section, we attempted to explain in detail our thematic analysis, performed by two independent reviewers with an inter-rater reliability of 0.69, which allowed us to gain deeper insight into the reasons and competing concerns driving student mental health burdens.

*4. I also have concerns regarding the applicability of the study tool among the stated study population.*

We aimed to address gaps in literature describing pandemic burdens on senior medical students who help to provide frontline care in the United States. Several of our authors are deans of medical schools, work closely with medical students, serve on Association of American Medical Colleges (AAMC) national committees and felt that this validated tool would be most useful and appropriate for the purposes of our study. Survey tool development involved consultation with stress psychology experts [1] and with psychiatric professionals on our own study team.We chose tools that were represented in the literature assessing pandemic impacts of SARS-CoV-1 and SARS-CoV-2 with medical student populations. PC-PTSD-5 has been used to assess pandemic impacts on healthcare providers [1, 24] and GAD-7 has been used both before and during this pandemic to assess well-being of medical students [3, 7-10, 12-20].

*5. Authors need to clearly state their study population in their manuscript.*

To clarify the study population given the variation in US medical school curricula, we have added Supplemental Table 2 with each school’s eligible student population based on their start of clinical rotations. The table includes the caption:

“Class years were based on the class in which a student started medical school, independent of any planned or unplanned time off; for an MD-PhD student starting in the class of 2021, for example, they were eligible if they had begun clinical rotations before April 2020 regardless of when they started their research time.”

*Reviewers' comments:*

*Reviewer's Responses to Questions*

*1. Is the manuscript technically sound, and do the data support the conclusions?*

*The manuscript must describe a technically sound piece of scientific research with data that supports the conclusions. Experiments must have been conducted rigorously, with appropriate controls, replication, and sample sizes. The conclusions must be drawn appropriately based on the data presented.*

*Reviewer #1: Partly*

*Reviewer #2: Partly*

We have addressed these concerns in our answers to (1)-(5) above. We have further clarified the quantitative and thematic analysis of our data, cited the use of the same validated tools in similar populations in the literature, removed references to mixed methods, and provided additional granular information about the breakdown of responses and cutoffs (Supplemental Table 3). While we do recognize in the manuscript that there are limitations, we believe that the uniqueness of the timeframe and the depth of analysis outweigh the limitations and that our data and conclusions offer an important contribution to characterizing the pandemic’s effects in the distinctive population of medical students in clinical training at the time of the initial peak.

*2. Has the statistical analysis been performed appropriately and rigorously?*

*Reviewer #1: No*

*Reviewer #2: No*

We have clarified the quantitative and thematic analyses performed and removed references to triangulation or mixed methods. We hope that these changes address concerns over our analyses and if reviewers request further clarifications, we are happy to revise.

*3. Have the authors made all data underlying the findings in their manuscript fully available?*

*The [PLOS Data policy](https://urldefense.com/v3/__http:/www.plosone.org/static/policies.action*sharing__%3BIw!!LQC6Cpwp!6VBgNhyv9pCqoxZwUaPoQ5nMV7bvhVIwnmlWLn1HRrtVpLCly_Blc0IvePQxGK03hyw$) requires authors to make all data underlying the findings described in their manuscript fully available without restriction, with rare exception (please refer to the Data Availability Statement in the manuscript PDF file). The data should be provided as part of the manuscript or its supporting information, or deposited to a public repository. For example, in addition to summary statistics, the data points behind means, medians and variance measures should be available. If there are restrictions on publicly sharing data—e.g. participant privacy or use of data from a third party—those must be specified.*

*Reviewer #1: No*

*Reviewer #2: Yes*

Per University of California, San Francisco policy, we are only able to publicly post our data after acceptance for publication. For ease of review, we are attaching the full dataset to this resubmission and we will be happy to publish it fully online if accepted.

*4. Is the manuscript presented in an intelligible fashion and written in standard English?*

*PLOS ONE does not copyedit accepted manuscripts, so the language in submitted articles must be clear, correct, and unambiguous. Any typographical or grammatical errors should be corrected at revision, so please note any specific errors here.*

*Reviewer #1: Yes*

*Reviewer #2: Yes*

*5. Review Comments to the Author*

*Please use the space provided to explain your answers to the questions above. You may also include additional comments for the author, including concerns about dual publication, research ethics, or publication ethics. (Please upload your review as an attachment if it exceeds 20,000 characters)*

*Reviewer #1: The setting under which the study was done i.e. during lockdown phase or when the students were sent back home or in hostel is not clear*

At the time the study was performed, students in United States medical schools had been pulled from their clinical rotations as per national recommendations [6, 8]. The absence of mandatory national COVID-19 policy in the United States makes it difficult to completely characterize local conditions; however, most states were under shelter-in-place orders. In the US medical system, student living quarters are highly variable, from apartments to school-sponsored dormitories to living with family. We captured data on students’ living situations and stratified our results based on their living environment (Table 2).

*What was the type of mixed methods design used -please mention*

As explained in responses to editor comments (1)-(5) above, we have removed references to any triangulation or mixed methods analysis.

*Analyzing a single open response question does not qualify this study as mixed methods design as no qualitative data collection technique has been used*

Thank you. After reconsideration, we have decided to simplify the analysis, remove the term “mixed methods,” and present thematic results without triangulating.

*Tabular representation of descriptive statistics of the items in the scale would have brought clarity*

Thank you so much for this suggestion; we have included an item-based data table as Supplemental Table 3.

*As it has been claimed that it was a mixed methods design, please mention the point of data integration/triangulation*

We have reconsidered this aspect of our analysis; please see above.

*Reviewer #2: Thank you for the opportunity to review this paper. In general, I think the study is limited by the low response rate, survey questions that may not have fully assessed the student experience, and a methodology which is limited in terms of being actually mixed.*

*My specific comments are as follows:*

*Abstract Methods: There is no mention of the population size to whom the survey was sent or the response rate.*

We describe in detail the population in which census sampling was used in the Methods section and provide additional context in Supplemental Table 2. We have added the phrase “Of 2511 eligible students, 741 responded (29.5%).**”**

*Introduction – Para 1, Sent 2 – psychological distress among medical students varies by year and may be less than in the general public at the start of medical school, making this sentence a bit of an over-generalization.*

Thank you. We have changed the wording to “medical student psychological distress varies by year, and is greater in clinical years” (Manuscript - Tracked Changes).

*Para 1, sent 4 - I'm not sure that the one study cited means that you can state that students who access MH resources show improvement in suicidality - My sense is that the summation of the literature regarding SI has shown equivocal efficacy of MH resources.*

Thank you very much for your detailed approach. We have changed the language to “have shown improvement” based on a current literature search that shows some benefit for medical students accessing mental health resources (Manuscript - Tracked Changes).

*In the 2nd and 3rd to last paragraphs of the introduction, the authors cite studies from populations across the world - the circumstances of the pandemic, cultural differences regarding mental health, and nature of medical student education are so different in all of those locations that it may not be best to rely on these studies for the background of a paper that is focused on US student populations.*

As discussed in our cover letter above, we agree that prevalence of anxiety and depression in US medical students cannot be assumed based on international student populations. However, we believe that this comment highlights the crucial gap in the literature that our study aims to address, and reinforces the appropriateness of the survey tools used. The international evidence of significant mental health effects of the pandemic in medical students also provides a reference point when considering the repercussions and specific stressors for US students.

*Methods, Para 1, Sent 2 – the authors refer to the choice of schools for the survey based “the” four different regions of the country. Did they use a specific rubric that divides the US into 4 well-established regions that are commonly used? From my estimation, there are indeed 4 regions represented, but others that are not. I would rephrase and also explain if some of the schools chosen were based on a convenience sample related to a pre-existing collaborative relationship of the co-authors.*

Schools were recruited intentionally to represent the conventional regions used to describe medical schools in the United States: West, South, East, and Midwest. For an example of this usage, see American Association of Medical Colleges’ “Virtual Medical School Fair.” 22 April 2021. <http://offers.aamc.org/Schools>. Additionally, we recruited schools specifically to represent variable levels of regional COVID-19 prevalence during the initial peak. Sampling required the establishment of novel collaborative relationships in each case and was not based on convenience.

*Please also indicate the medical student year of included participants - were they all MS3s and MS4s, or were some of the invited students MS2s, MS5s who had taken a scholarly year, or perhaps MD-PhD students who had been students for even longer, as this variable may influence the findings.*

We collected data from participants based on when they had started clinical rotations by asking about their intended graduation year, exclusive of any time off. This means that only students who would have graduated in 2021, 2020, or 2022 (regardless of MD-PhD status) were included. Because there is indeed variation in graduation timelines, after careful consideration we defined these based on the start of rotations, and therefore students’ likely exposure to the clinical environment prior to the pandemic, and not the intended finish. This method helped us narrow our sample to the population of interest, medical students who had had clinical roles prior to or at the time of the start of the pandemic. Please see Supplemental Table 2 for additional information about each school’s timeline.

*Survey Instrument - why were the questions used to elicit pandemic-related concerns taken from a study of EM physicians? It would seem that the experience of EM physicians would be quite different from that of medical students? If there was no compelling reason to use these EM MD-related questions, why not use questions more closely designed to examine the student experience, even if not validated or previously published?*

Thank you for this question. We used only relevant questions from the EM physician survey and modified them for appropriateness to the medical student population. The validated instruments that were used in both surveys have also been used in many other healthcare populations [24]. We have added the phrase in Methods, “modified to represent our study population.” As the previously published portion of our data shows, senior clinical medical students have been involved in the frontline healthcare workforce to varying extents during this pandemic, and we believe tools used to measure mental health impacts on healthcare workers are therefore appropriate [8].

*Analysis - it's not clear from the text if multivariable analyses were conducted.*

Our stratification tables present univariate analyses, as was most appropriate for the response scales we analyzed.

*Results – the response rate is quite low – do you have any data on non-respondents to determine if your sample is reflective of the larger population?*

Unfortunately, due to the disruptions of the initial peak of the pandemic (including early graduation at half of the schools surveyed), we do not have data on non-respondents. We have addressed both the limitations of our response rate and similar response rates seen during the pandemic with survey tools in the paper. Other widely-cited studies of acute mental health impacts have also shown diminished response rates, suggesting the limits of survey tools during times of large-scale economic and social disruption [5].

*Discussion - Paragraphs 1 and 2 and 3. The findings of the study - it seems that the major findings of the quantitative aspects of the survey mirror largely what has been found in the general population, which may in part be due to the lack of inclusion of questions that more specifically ascertain the impact of the pandemic on medical student-related drivers of stress. Some of this is captured in the qualitative component of the study, though, if I understand correctly, the qualitative component consists of free text questions on a survey, as opposed to more commonly used qualitative methodologies such as focus groups and interviews. The study would have been strengthened by the usage of such methodologies and the inclusion of survey questions that could drill down to assess stressors more specifically related to being a student. This is captured to some extent in paragraph 4 and I would expand upon that if possible.*

We very much appreciate and share your interest in this question. Our prior study published a cross-sectional analysis of the specific academic impacts of the pandemic on clinical medical students [8]. In this manuscript, we have included our analysis of questions that addressed the specific drivers of student stress and anxiety during this period. Figure 1 shows students’ responses to Likert questions assessing their level of concern for personal safety, community safety, and the safety and capacity of their professional teams and healthcare systems. Table 3 also presents themes that give a nuanced picture of concerns in students’ own words. As we describe in the Discussion, we are hopeful that this data will serve as encouragement to schools to use the qualitative methods you mention to fully explore their students’ needs as we move forward in the pandemic.

*Limitations - preserving and minimizing survey length should not be used as an explanation for why depression and suicidality were not included as outcomes as very short item screens exist to assess these outcomes, just 1-2 questions long in some instances.*

Thank you, we agree and have made the following change:

“The scope of this study focused on anxiety and PTSD specifically; there are likely other mental health burdens that were not assessed, such as suicidality.”

**S2 Table. Eligible clinical student populations by medical school**

| School | Classes Eligible | N |
| --- | --- | --- |
| University of California, San Francisco* | 2020  2021  2022 | 162  186  208 |
| University of California, Irvine⧫ | 2020  2021 | 104  104 |
| University of Illinois⧫ | 2020  2021 | 316  334 |
| Ohio State University⧫ | 2021  2022 | 201  209 |
| Tulane University⧫ | 2020  2021 | 195  191 |
| Hofstra University* | 2020  2021  2022 | 99  101  101 |

*Schools which begin clinical rotations in the second year of medical school

⧫Schools beginning clinical rotations in the third year of medical school

Curricula vary by medical school in the United States. Students in clinical years were deemed eligible for the survey if they had begun clinical rotations by the time of survey administration. Class years were based on the class that a student started medical school in, independent of any planned or unplanned time off; for an MD-PhD student starting in the class of 2021, for example, they were eligible if they had begun clinical rotations before April 2020 regardless of when they started their research time.

**S3 Table. Descriptive statistics for anxiety and PTSD scales**

| GAD-7  How often have you been bothered by the following over the past 2 weeks: | Not at all  N (%) | Several days  N (%) | More than half the days  N (%) | Nearly every day  N (%) |
| --- | --- | --- | --- | --- |
| Feeling nervous, anxious or on edge? | 191 (25.9%) | 310 (42.1%) | 155 (21.0%) | 81 (11.0%) |
| Not being able to stop or control worrying? | 323 (43.8%) | 250 (33.9%) | 103 (14.0%) | 62 (8.4%) |
| Worrying too much about different things? | 223 (30.3%) | 285 (38.7%) | 136 (18.5%) | 93 (12.6%) |
| Trouble relaxing? | 234 (31.9%) | 286 (39.0%) | 134 (18.3%) | 80 (10.9%) |
| Being so restless that it's hard to sit still? | 436 (59.4%) | 174 (23.7%) | 82 (11.2%) | 42 (5.7%) |
| Becoming easily annoyed or irritable? | 244 (33.0%) | 305 (41.3%) | 125 (16.9%) | 65 (8.8%) |
| Feeling afraid as if something awful might happen? | 402 (54.4%) | 210 (28.4%) | 84 (11.4%) | 43 (5.8%) |
|  | | | | |
| Score Range | None (0-4) | Mild (5-9) | Moderate (10-14) | Severe (15-21) |
|  | 292 (39.6%) | 253 (34.3%) | 119 (16.1%) | 70 (9.5%) |

| PC-PTSD-5  Since the start of the COVID-19 pandemic, have you: | Yes  N (%) |
| --- | --- |
| Had nightmares related to the pandemic or thought about the pandemic when you did not want to? | 230 (31.1%) |
| Tried hard not to think about the pandemic or gone out of your way to avoid situations that reminded you of it? | 298 (40.4%) |
| Been constantly on guard, watchful, or easily startled? | 168 (22.8%) |
| Felt numb or detached from people, activities or your surroundings? | 253 (34.4%) |
| Felt guilty or unable to stop blaming yourself or others for the effects of the pandemic or any problems the pandemic may have caused? | 152 (20.6%) |
|  | |
| At least 3 “yes” | 188 (25.4%) |
| At least 4 “yes” | 86 (11.6%) |
